# Previously Identified Genetic Variants in *ADGRL3* Are not Associated with Risk for Equine Degenerative Myeloencephalopathy across Breeds

**DOI:** 10.3390/genes10090681

**Published:** 2019-09-05

**Authors:** Sabin A. Marquardt, Callie V. Wilcox, Erin N. Burns, Janel A. Peterson, Carrie J. Finno

**Affiliations:** Department of Population, Health and Reproduction at the University of California, Davis, Davis, CA 95616, USA (S.A.M.) (C.V.W.) (E.N.B.) (J.A.P.)

**Keywords:** equine neuroaxonal dystrophy, horse, vitamin E

## Abstract

Equine neuroaxonal dystrophy/equine degenerative myeloencephalopathy (eNAD/EDM) is a neurologic disease that has been reported in young horses from a wide range of breeds. The disease is inherited and associated with vitamin E deficiency during the first two years of life, resulting in bilateral symmetric ataxia. A missense mutation (chr3:71,917,591 C > T) within adhesion G protein-coupled receptor L3 (*ADGRL3*) was recently associated with risk for EDM in the Caspian breed. In order to confirm these findings, genotyping of this missense mutation, along with the three other associated single nucleotide polymorphisms (SNPs) in the genomic region, was carried out on 31 postmortem-confirmed eNAD/EDM cases and 43 clinically phenotyped controls from various breeds. No significant association was found between eNAD/EDM confirmed cases and genotype at any of the four identified SNPs (*P* > 0.05), including the nonsynonymous variant (EquCab2.0 chr3:71,917,591; allelic *P* = 0.85). These findings suggest that the four SNPs, including the missense variant in the *ADGRL3* region, are not associated with risk for eNAD/EDM across multiple breeds of horses.

## 1. Introduction

Equine neuroaxonal dystrophy/equine degenerative myeloencephalopathy (eNAD/EDM) is a neurologic disease that has been reported in young horses from a wide range of breeds. Equine degenerative myeloencephalopathy (EDM) is a more histologically advanced form of equine neuroaxonal dystrophy (eNAD). Equine NAD/EDM has been associated with vitamin E deficiency during the first two years of life [1], resulting in the development of bilaterally symmetric proprioceptive ataxia [2,3]. Using immunohistochemistry, deficits in the ascending proprioceptive tracts of the spinal cord to the originating cell bodies within the dorsal root ganglion (DRG) were traced [4]. It has been hypothesized that eNAD/EDM has either an autosomal dominant or polygenic mode of inheritance [5,6,7]. However, an autosomal recessive inheritance pattern has also been proposed [8]. Diagnosis of eNAD/EDM can only be confirmed by postmortem histological evaluation of the brainstem and spinal cord [3]. Four single nucleotide polymorphisms (SNPs) were recently identified to be associated with EDM in the Caspian horse breed [8]. These variants were within adhesion G protein-coupled receptor L3 (*ADGRL3*) and the nonsynonymous variant was proposed to be associated with risk for EDM in this breed [8]. Of the four SNPs, SNP 1 (EquCab2.0 chr3:71,768,217 T > A) is located within the 3′ untranslated region of *ADGRL3*, SNP 2 (EquCab2.0 chr3:71,770,084 T > C; synonymous) and SNP 4 (chr3:71,917,591 C > T; non-synonymous) are exonic, and SNP 3 (EquCab2.0 chr3:71,836,145 G > A;) is intronic. As the putative variant was identified using two related EDM-affected Caspian horses and there was a lack of appropriate controls [8], we hypothesized that this previously reported *ADGRL3* variant would not be associated with eNAD/EDM status across breeds.

## 2. Materials and Methods

A total of 74 horses, 31 eNAD/EDM cases and 43 controls, were used in this study. Affected horses were selected based on their postmortem diagnosis by board-certified veterinary pathologists. Control horses were all over the age of 6 months and either postmortem-confirmed (*n* = 13) as unaffected or known to be vitamin E-deficient during the first few years of life, though they demonstrated no neurologic abnormalities when examined as adults (*n* = 30). The affected cohort included 14 Quarter Horses, four Warmbloods, two Andalusians, two Thoroughbreds, two Dutch Warmbloods, two Lusitanos, and one each of Paint, Morgan, Fell Pony, Thoroughbred/Quarter Horse Cross, and Arabian. The unaffected cohort included 21 Quarter Horses, seven Warmbloods, five Thoroughbreds, two Lusitanos, two Paints, and one each of Morgan, Thoroughbred/Quarter Horse Cross, Arabian, Percheron, Friesian, and Pony of America. The disease status, ages, sexes, breeds, and genotypes of these horses can be found in Supplementary Table S1. The cases had an average age of 2.8 years, while the average age of the control horses was 9.6 years.

From each horse, a sample of genomic DNA was isolated from blood or tissue using the Promega Wizard gDNA kit (Promega, Madison, WI, USA) or Gentra Puregene kit (Qiagen, Germantown, MD, USA). Primer3Plus [9] was used to design four primer sets to amplify the regions containing one of the four SNPs of interest (EquCab2.0; chr3:71,768,217 T > A, chr3:71,770,084 T > C, chr3:71,836,145 G > A, chr3:71,917,591 C > T). These primer sets differed from the sets previously reported [8]. Primer sequences and annealing temperatures can be found in Supplementary Table S2. Samples were randomized for genotyping. Polymerase chain reaction (PCR) was performed and verified by gel electrophoresis, then ExoSap-IT™ PCR Cleanup Reagent (Thermo Fisher Scientific, Waltham, MA, USA) was used according to manufacturer’s recommendations. Sanger sequencing was performed and the sequences were analyzed using Sequencher (Gene Codes Corporation, Ann Arbor, MI, USA). A Fisher’s Exact Test was performed for each SNP using both allelic counts and genotypic counts and significance was evaluated using both an uncorrected (*P* < 0.05) and Bonferroni-corrected (*P* < 0.012) *P* value. The Functional Annotation of the Animal Genome (FAANG) alignment files for Equcab2.0 were viewed using Integrative Genome Viewer (IGV) [10] to analyze *ADGRL3* expression in the frontal lobe, occipital lobe, parietal lobe, cerebellum, lumbar spinal cord, and dorsal root ganglia. SNP positions were converted to EquCab3.0 using the NCBI remap tool (https://www.ncbi.nlm.nih.gov/genome/tools/remap) for comparison. 

## 3. Results

**Figure 1:**
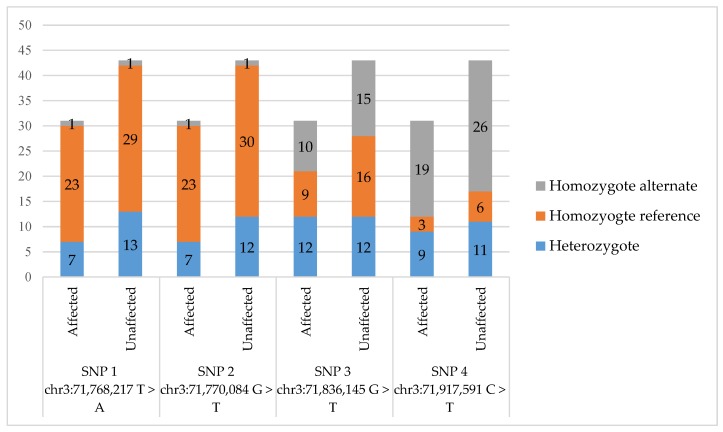
Graphical representation of the genotype distribution of all genotyped horses. The number of horses is denoted in each section of the bar graph. Single nucleotide polymorphism (SNP)positions are from EquCab2.0. No SNP achieved a significant (*P* < 0.05) association with the equine neuroaxonal dystrophy/equine degenerative myeloencephalopathy (eNAD/EDM) phenotype.

Individual horse genotypes for each SNP are provided in Supplementary Table S1. For SNP 1 (chr3:71,768,217 T > A), only two homozygous alternate individuals were found in the study population, one case and one control. Seven cases and 13 controls were heterozygous, and the remaining 23 cases and 29 controls were homozygous for the reference allele (allelic *P* = 0.66) (Figure 1). Similar to SNP 1, only two homozygous alternate individuals were found for SNP 2 (chr3:71,770,084 T > C), along with seven heterozygous cases and 12 heterozygous controls (allelic *P* = 0.82) (Figure 1). For SNP 3 (chr3:71,836,145 G > A), 10 cases and 15 controls were homozygous for the alternate allele, with nine cases and 16 controls being homozygous for the reference allele and 12 cases and controls being heterozygous (allelic *P* = 0.87) (Figure 1). Finally, genotyping of SNP 4 (chr3:71,917,591 C > T) identified 19 cases and 26 controls as homozygous for the alternate genotype. Only 12 cases and 17 controls contained the reference allele (allelic *P* = 0.85) (Figure 1). There was no significant difference (*P* > 0.05) in proportion of homozygous reference genotype, homozygous alternate genotype, and heterozygous individuals between cases and controls across the four SNPs (Figure 1). A Fisher’s Exact Test was also performed to compare genotypes since a recessive mode of inheritance has been suggested [8], resulted in *P* = 1.00 for all SNPs assuming a recessive mode of inheritance, demonstrating no association between the homozygous recessive genotype and eNAD/EDM cases. Remapping of SNP positions to EquCab3.0 (Supplementary Table S3) revealed a change in the reference allele for the chr3:71,836,145 SNP. The new reference was A and the alternate G at EquCab3.0 chr3:73,700,220.

## 4. Discussion

These data collected in this study fail to support an association between the four previously identified SNPs in *ADGRL3* and eNAD/EDM cases. It should also be noted that the two controls in the previous study were half-siblings and therefore relatedness could be the cause of associated genotype findings [8]; a concern that has been raised previously [11]. Within our study, there were no closely related individuals. As there were no Caspian horses in the current study sample, we are unable to draw conclusions regarding the Caspian breed. However, the Caspian breed is closely related to the Middle Eastern breeds, such as Arabian and Akhal Teke [12]. Though located on different branches of an unrooted neighbor joining tree, the Middle Eastern breeds are also related to the Iberian breeds, which include Andalusian and Lusitano [12]. Our study included one Arabian, one Lusitano, one Lusitano cross, and three Andalusians that were affected, as well as one Arabian and two Lusitanos that were unaffected (Supplementary Table S1). All affected and unaffected Arabians, Andalusians, and Lusitanos were the homozygote reference genotype for SNP 1 (chr3:71,768,217 T > A) and SNP 2 (chr3:71,770,084 T > C) (Supplementary Table S1). For SNP 3 (chr3:71,836,145 G > A), affected and unaffected Arabian and Lusitano horses were either the homozygote reference or heterozygote genotype (Supplementary Table S1). The affected and unaffected Lusitano horses were the homozygote alternate genotype or the heterozygote genotype for SNP 4 (chr3:71917591 C > T) (Supplementary Table S1). Therefore, the four variants were determined to be not associated with eNAD/EDM, even within the Arabian and Lusitano breeds. Additionally, as the alternate alleles for each SNP were identified across many breeds, these variants are unlikely to have a detrimental effect. 

Although expressed in the central nervous system, *ADGRL3* is not a strong candidate gene for eNAD/EDM. Equine NAD/EDM is associated with a postnatal vitamin E deficiency and most closely resembles ataxia with vitamin E deficiency in humans, an inherited disorder with mutations in tocopherol transfer protein (*TTPA*) [5]. The current literature does not support a role for *ADGRL3* in vitamin E absorption, transport, or metabolism. Moreover, *ADGRL3* is not expressed in intestinal or hepatic tissue (https://www.genecards.org/cgi-bin/carddisp.pl?gene=ADGRL3). Instead, *ADGRL3* genetic variants have been associated with attention-deficit/hyperactivity disorder (ADHD) and autism spectrum disorder (ASD) susceptibilities [13,14]. *ADGRL3* is highly expressed in human fetal brain and spinal cord (https://www.genecards.org/cgi-bin/carddisp.pl?gene=ADGRL3) and horses demonstrate a similar pattern of gene expression throughout the frontal lobe, occipital lobe, parietal lobe, cerebellum, lumbar spinal cord, and dorsal root ganglia [15] (https://www.ebi.ac.uk/ena/data/view/ERA148755). However, in horses with eNAD/EDM, axonal swellings, termed spheroids, occur in the brainstem and spinal cord [4]. There has been no association, in any species, with genetic *ADGRL3* variants leading to axonal loss. 

In conclusion, we have demonstrated no genetic association between previously identified genetic variants in *ADGRL3* and eNAD/EDM across many breeds of horses. As this population of horses was stringently phenotyped using postmortem-confirmed cases and a large number of postmortem-confirmed controls, and the alternate allele for these variants was found in many breeds, these variants should not be used when diagnosing ataxic horses for eNAD/EDM or to inform breeding decisions. 

## Supplementary Materials

The following are available online at https://www.mdpi.com/2073-4425/10/9/681/s1, Table S1: All individuals included in the current study with their disease status, respective age (at euthanasia or exam), sex, breed, and genotype at each SNP. Table S2: Primers and annealing temperatures for each SNP genotyped. Table S3: SNP positions in EquCab2.0 compared to EquCab3.0.
