# Supplementary material for: Previously Identified Genetic Variants in ADGRL3 Are not Associated with Risk for Equine Degenerative Myeloencephalopathy across Breeds
Source: Genes (Basel). 2019 Sep 5;10(9):681. doi: 10.3390/genes10090681 (PMC6770705; doi:10.3390/genes10090681)
Supplement: Supplementary file 1 [file genes-10-00681-s001.pdf]

**Table S1.** All individuals included in the current study with their disease status, respective age (at euthanasia or exam), sex, breed, and genotype at each SNP. SNP positions are from EquCab2.0. A:PM=Affected Postmortem confirmed, U:PM=unaffected postmortem confirmed, U=unaffected clinically confirmed. ?=Unknown. S=Stallion, G=Gelding, M=Mare, FP=Female Pseudo. QH=Quarter Horse, WB=Warmblood, DWB=Dutch Warmblood, POA=Pony of the Americas, TB=Thoroughbred.

| Status | Age (years) | Sex | Breed | SNP 1 (chr3:71,768,217 T > A) genotype | SNP 2 (chr3:71,770,084 T > C) genotype | SNP 3 (chr3:71,836,145 G > A) genotype | SNP 4 (ch3:71,917,591 C > T) genotype |
|---|---|---|---|---|---|---|---|
| A: PM | 0.5 | M | Andalusian | T/T | T/T | A/G | T/T |
| A: PM | 6 | G | Andalusian | T/T | T/T | A/G | T/T |
| A: PM | 5 | G | Andalusian | T/T | T/T | G/G | T/T |
| A: PM | 7 | G | Arabian | T/T | T/T | A/G | C/T |
| A: PM | 1 | M | DWB | T/T | T/T | A/G | C/T |
| A: PM | 1 | M | DWB | A/T | C/T | A/A | T/T |
| A: PM | 11 | S | Fell Pony | T/T | T/T | A/G | C/C |
| A: PM | 1 | M | Lusitano | T/T | T/T | G/G | C/T |
| A: PM | 0.5 | S | Morgan | A/T | C/T | A/A | T/T |
| A: PM | 2 | G | Paint | T/T | T/T | G/G | T/T |
| A: PM | 1 | S | QH | T/T | T/T | A/A | T/T |
| A: PM | 1 | S | QH | A/A | C/C | A/A | T/T |
| A: PM | 3 | M | QH | T/T | T/T | G/G | C/C |
| A: PM | 1 | G | QH | T/T | T/T | A/G | T/T |
| A: PM | 1 | S | QH | A/T | C/T | A/A | T/T |
| A: PM | 4 | G | QH | T/T | T/T | A/A | T/T |
| A: PM | 7 | G | QH | T/T | T/T | G/G | C/C |
| A: PM | 1 | G | QH | A/T | C/T | A/G | T/T |
| A: PM | 2 | G | QH | A/T | C/T | A/G | T/T |
| A: PM | 1.5 | M | QH | T/T | T/T | A/A | T/T |
| A: PM | 1.5 | M | QH | T/T | T/T | A/A | T/T |
| A: PM | 4 | G | QH | A/T | C/T | A/G | T/T |

| | | | | | | | |
|---|---|---|---|---|---|---|---|
| A: PM | 4 | G | QH | T/T | T/T | A/A | T/T |
| A: PM | 0.6 | M | QH | T/T | T/T | G/G | T/T |
| A: PM | 1 | S | QH/Lusitano | T/T | T/T | A/G | C/T |
| A: PM | 2 | S | TB | T/T | T/T | G/G | C/T |
| A: PM | 2 | S | TB | T/T | T/T | A/G | C/T |
| A: PM | 4 | G | TB/QH | T/T | T/T | G/G | C/T |
| A: PM | 2 | G | WB | A/T | C/T | A/A | T/T |
| A: PM | 3 | G | WB | T/T | T/T | A/A | T/T |
| A: PM | 4 | M | WB | T/T | T/T | A/G | C/T |
| U: PM | 18 | M | Paint | A/T | C/T | A/A | C/C |
| U: PM | 0.5 | FP | Percheron | T/T | T/T | G/G | T/T |
| U: PM | 1 | M | POA | T/T | T/T | A/G | C/T |
| U: PM | 14 | M | QH | A/T | C/T | A/A | T/T |
| U: PM | 28 | M | QH | A/T | T/T | A/G | T/T |
| U: PM | 2 | G | QH | T/T | T/T | G/G | C/T |
| U: PM | 1 | M | QH | T/T | T/T | G/G | C/T |
| U: PM | 6 | M | QH | A/T | C/T | A/A | T/T |
| U: PM | 34 | M | QH | T/T | T/T | A/A | C/T |
| U: PM | 2 | M | QH | A/T | C/T | A/A | C/C |
| U: PM | 25 | G | QH | A/T | C/T | A/A | T/T |
| U: PM | 13 | S | QH | T/T | T/T | A/A | T/T |
| U: PM | 5 | G | TB | T/T | T/T | G/G | C/C |
| U | ? | M | Arabian | T/T | T/T | A/G | C/C |
| U | 9 | M | DWB | T/T | T/T | A/G | C/T |
| U | 5 | S | Friesian | T/T | T/T | G/G | T/T |
| U | 1 | S | Lusitano | T/T | T/T | G/G | C/T |
| U | 1 | M | Lusitano | T/T | T/T | A/A | T/T |
| U | 7 | M | Morgan | A/T | A/T | A/G | C/T |

| | | | | | | | |
|---|---|---|---|---|---|---|---|
| U | 8 | M | Paint | T/T | T/T | G/G | T/T |
| U | 11 | G | QH | T/T | T/T | A/A | C/T |
| U | 18 | M | QH | A/T | C/T | A/G | T/T |
| U | 12 | M | QH | A/A | C/C | A/A | T/T |
| U | 6 | G | QH | T/T | T/T | G/G | T/T |
| U | 8 | M | QH | A/T | C/T | A/G | T/T |
| U | 8 | M | QH | A/T | C/T | A/G | T/T |
| U | 9 | M | QH | A/T | C/T | A/G | T/T |
| U | 7 | S | QH | T/T | T/T | G/G | T/T |
| U | 19 | M | QH | T/T | T/T | A/A | T/T |
| U | 9 | M | QH | T/T | T/T | G/G | T/T |
| U | 8 | S | QH | A/T | C/T | A/A | T/T |
| U | 8 | S | QH | A/T | C/T | A/A | T/T |
| U | 9 | S | QH/TB | T/T | T/T | G/G | C/T |
| U | 8 | M | TB | T/T | T/T | A/G | C/T |
| U | 7 | M | TB | T/T | T/T | A/A | T/T |
| U | 8 | M | TB | T/T | T/T | A/A | T/T |
| U | 15 | S | TB | T/T | T/T | A/G | C/C |
| U | 12 | G | WB | T/T | T/T | G/G | C/T |
| U | 6 | M | WB | T/T | T/T | GG | T/T |
| U | 13 | M | WB | T/T | T/T | G/G | C/T |
| U | 9 | M | WB | T/T | T/T | G/G | T/T |
| U | 5 | M | WB | T/T | T/T | G/G | C/C |
| U | 8 | G | WB | T/T | T/T | G/G | T/T |

**Table S2.** Primers and annealing temperatures for each SNP genotyped.

| SNP Coordinate | Sequence | Annealing Temp. (ºC) | Product Length (bp) |
|---|---|---|---|
| Chr3:71,768,217 | F-AGTAGGATTCGACCCCTCCC | 64.5 | 706 |
| | R-GAATGCTCAGTCTGACCCCC | 64.5 | |

| | | | |
|---|---|---|---|
| Chr3:71,770,084 | F-TCTGGAGGGGTCCCATCTTT | 62.4 | 753 |
| | R-GCTTCTGAACAATGCCAGGG | 62.4 | |
| Chr3:71,836,145 | F-AGTTCATCCAGATCCAGAAGCT | 60.8 | 750 |
| | R-ATTAGGGCCATGTGCAGAGG | 62.4 | |
| Chr3:71,917,591 | F-TATAGTTCCTGCAGGGCACG | 62.4 | 717 |
| | R-TCCCCAGTTTGCACCATCTT | 60.4 | |

**Table S3.** SNP positions in EquCab2.0 compared to EquCab3.0. The reference allele for the SNP in red changes in EquCab3.0. For this SNP reference in EquCab3.0 is A while alternate is G. Ref=Reference. Alt=Alternate.

| EquCab2.0 | EquCab3.0 | Ref. (2.0) | Alt. |
|---|---|---|---|
| chr3:71,768,217 | chr3:73,632,279 | T | A |
| chr3:71,770,084 | chr3:73,634,146 | T | C |
| chr3:71,836,145 | chr3:73,700,220 | G | A |
| chr3:71,917,591 | chr3:73,781,733 | C | T |
